# Prostaglandin insert dinoprostone versus trans-cervical balloon catheter for outpatient labour induction: a randomised controlled trial of feasibility (PROBIT-F)

**DOI:** 10.1186/s40814-020-00661-7

**Published:** 2020-08-15

**Authors:** Amarnath Bhide, Philip Sedgwick, Barbara Barrett, Georgina Cupples, Rose Coates, Rosie Goode, Sandra Linton, Christine McCourt

**Affiliations:** 1 Fetal Medicine Unit, St. George’s University Hospital Foundation Trust, Blackshaw Road, London, SW17 0QT UK; 2 St George’s, University of London, London, UK; 3 UiT: The Arctic University of Norway, Tromsø, Norway; 4 King’s Clinical Trials Unit, London, UK; 5 City, University of London, London, UK; 6 Maternity Voices Partnership Chair, St. George’s University Hospital Foundation Trust, London, UK

**Keywords:** Labour, induced, Cervical ripening, Randomised controlled trial, Cook cervical ripening balloon, Outpatients, feasibility

## Abstract

**Background:**

The aim was to assess the feasibility of conducting a randomised controlled trial (RCT) of induction of labour comparing use of two methods in the outpatient setting.

**Methods:**

An open-label feasibility RCT was conducted in two UK maternity units from October 2017 to March 2019. Women aged ≥ 16 years, undergoing induction of labour (IOL) at term, with intact membranes and deemed suitable for outpatient IOL according to local guidelines were considered eligible. They were randomised to cervical ripening balloon catheter (CRB) or vaginal dinoprostone (Propess). The participants completed a questionnaire and a sub-group underwent detailed interview. Service use and cost data were collected via the Adult Service Use Schedule (AD-SUS). Women who declined to participate were requested to complete a decliners’ questionnaire.

**Results:**

During the study period, 274 eligible women were identified. Two hundred thirty (83.9%) were approached for participation of whom 84/230 (36.5%) agreed and 146 did not. Of these, 38 were randomised to Propess (*n* = 20) and CRB (*n* = 18). Decliner data were collected for 93 women. The reasons for declining were declining IOL (*n* = 22), preference for inpatient IOL (*n* = 22) and preference for a specific method, Propess (*n* = 19). The intended sample size of 120 was not reached due to restrictive criteria for suitability for outpatient IOL, participant preference for Propess and shortage of research staff.

The intervention as randomised was received by 29/38 (76%) women. Spontaneous vaginal delivery was observed in 9/20 (45%) women in the dinoprostone group and 11/18 (61%) women in the CRB group. Severe maternal adverse events were recorded in one woman in each group. All babies were born with good condition and all except one (37/38, 97.4%) remained with the mother after delivery. No deaths were recorded. − 21% of women in the dinoprostone group were re-admitted prior to diagnosis of active labour compared to 12% in the CRB group.

**Conclusions:**

A third of the approached eligible women agreed for randomisation. An RCT is not feasible in the current service context. Modifications to the eligibility criteria for outpatient IOL, better information provision and round the clock availability of research staff would be needed to reach sufficient numbers.

**Trial registration:**

NCT03199820. Registered on June 27, 2017

## Key messages

- The study is not feasible using existing eligibility criteria for outpatient induction of labour (IOL); further modifications to the eligibility criteria for outpatient IOL would be needed to make it feasible.
- Other reasons for the low recruitment rate were participant preference for prostaglandin pessary and shortage of research staff.
- No major adverse effects were recorded attributible to the outpatient setting for induction of labour in this small sample.

## Introduction

Over the last decade, the rate of induction of labour (IOL) in England has increased steadily to around 30% of all pregnant women [1]. Currently, most women undergoing induction of labour are admitted to the hospital prior to commencing IOL. A Cochrane review assessing methods of outpatient labour induction (cervical ripening or priming) concluded that it was feasible for labour to start at home. However, there is limited evidence as to which induction methods are preferred by women or the interventions that are most effective and safe to use in outpatient settings [2]. A Cochrane review reported that mechanical methods (trans-cervical balloon catheter) of cervical ripening for IOL are as effective as vaginal prostaglandin PGE_2_ [3]. The UK Database of Uncertainties about the Effects of Treatments (UK DUETs) identified mechanical methods of labour induction as a known uncertainty and recommended that future studies on mechanical methods for IOL should have larger sample sizes and report on substantive outcomes. In a randomised controlled trial [4], 101 women with an unfavourable cervix requiring IOL at term were randomised to outpatient care using Foley catheter or inpatient care using vaginal PGE_2_. The authors reported that the outpatient group had shorter hospital stay prior to birth whilst vaginal birth rates, total induction to delivery time and total inpatient times were similar. Another trial showed that, for women with an unfavourable cervix at term, success of induction of labour with a mechanical method is similar to induction of labour with progstaglandins, with fewer maternal and neonatal side effects, but similar caesarean section rates [5]. Furthermore, Pennell et al. [6] reported lower pain scores with the use of mechanical method as compared to prostaglandins. Both studies were apparently undertaken in an inpatient setting. The OPRA study [7] compared clinical outcomes from outpatient with inpatient prostaglandin treatment for low risk labour induction. They concluded that uterine stimulation following prostaglandins may preclude a woman from going home or remaining at home overnight and may not be the best agent for outpatient ripening. Therefore, it would be beneficial to compare outpatient outcomes of prostaglandin treatment with mechanical methods including economic analysis to determine the most suitable agent. The prostaglandin method is the standard practice for IOL at St. George’s Hospital, London, and Medway Hospital, Kent. Although mechanical methods are used in some UK hospitals, outpatient use is not common. The Hospital Episode Statistics (HES) database does not record the exact method of induction of labour nor collect data on efficacy, cost-effectiveness, hospital stay or outcome of labour induction stratified according to the method of induction of labour. Therefore, there is no readily available data source that can be used to obtain information on the outcomes of induction of labour using mechanical methods in the outpatient setting. A recent Cochrane review [3] concluded that future research could be focused more on safety aspects for the neonate and maternal satisfaction.

A feasibility trial was deemed necessary before embarking on a randomised controlled trial. It would permit identification of suitable clinical outcome measures with sufficient precision and help design a future randomised controlled trial. The primary objective, therefore, was to investigate the feasibility of conducting a randomised controlled trial of induction of labour using trans-cervical ballon catheter versus vaginal prostaglandin E_2_ pessary in the outpatient setting.

## Methods

### Study design

We conducted an open-label feasibility RCT (Registration Number: NCT03199820) with sustained-release prostaglandin vaginal pessary (Propess) or cervical ripening balloon catheter (CRB) in the outpatient setting using a 1:1 allocation ratio. The trial took place in two UK maternity units: St George’s University Hospitals NHS Foundation Trust, South London (October 2017 to March 2019) and Medway University Hospital, Kent (February 2018 to October 2018). The two sites differed in social demographics and were included to enhance the external validity of the results. The study description is publicly available at the clinical trials registry [8]. The trial was approved by the East of England - Cambridgeshire and Hertfordshire Research Ethics Committee (17/EE/0295). The primary objective was to assess the feasibility of conducting a randomised controlled trial (RCT), namely, the number of women willing to enrol. Secondary objectives were to identify suitable clinical outcome measures, estimate service costs and monitor safety, as well as to determine women’s willingness to be randomised, to determine the acceptability of using the balloon catheter, to examine women’s views on outpatient induction of labour and to assess women’s experience with these methods and their preference. Assessment of women’s experience with these methods and their preference was through interviews and qualitative analysis and will be reported elsewhere.

### Participants

Inclusion criteria were women aged ≥ 16 years, undergoing IOL at ≥ 37 weeks’ gestation, with intact membranes, able to give informed consent and deemed suitable for outpatient IOL according to local guidelines. Written information was provided to women regarding the available methods: IOL with sustained release dinoprostone (Propess), or cervical ripening balloon (CRB), both in the outpatient setting. Research teams at each site approached women to confirm eligibility and provided verbal and written information. Strict eligibility criteria have been developed for suitability of outpatient IOL against which research midwives screened for eligible participants and a medical practitioner confirmed that eligibility was met. At both the recruiting sites, the pregnancy had to be uncomplicated at or beyond 41^+0^ weeks with a single foetus in cephalic presentation with no risk factors. Trained clinician obtained written informed consent. During the study, the investigators noticed a shortfall of eligible women. Therefore, inclusion criteria were widened in 2018 at St. George’s Hospital to include women requiring induction of labour at term (37^+0^ weeks or more) with diet-controlled gestational diabetes, who were originally excluded.

### Randomisation and masking

Eligible participants were randomly allocated with a 1:1 ratio to receive Propess or CRB. Randomisation was stratified by site and parity using variable block sizes (two and four). A research team member entered baseline data on a web-based database at study enrolment and then allocated the treatment (Propess or CRB) using the web-based randomisation programme developed by the King’s Clinical trial Unit (KCTU). Nature of the intervention mandated that trial participants, clinical care providers or outcome assessors could not be blinded to trial allocation. The statistician was not aware of the allocation sequence and discussions as regards recruitment and was not involved in any of the women’s care or recording of their results. The data were supplied to the statistician by the Clinical Trials Unit with the group allocation coded. The group allocation was only revealed following compilation of the results in tabular form.

### Procedures

After randomisation, a member of the research team administered the treatment method according to the recommendations of the manufacturer, described briefly as follows: for induction of labour with Propess, 10 mg insert was introduced in the posterior vaginal fornix close to the cervix, using only small amounts of water-soluble lubricants to aid insertion. The woman was advised to be recumbent for 20-30 min following insertion. For IOL with balloon catheter, the woman was positioned in the dorsal position and a vaginal speculum was inserted to gain cervical access. The cervix was cleaned appropriately to prepare for device insertion. The CRB was inserted into the cervix and advanced until both balloons entered the cervical canal. The uterine balloon was inflated with 40 ml sodium chloride (0.9%). Once the uterine balloon was inflated, the device was pulled back until the balloon was against the internal cervical os. The vaginal balloon was now inflated with 20 ml NaCl (0.9%). The speculum was removed after the balloons were situated on each side of the cervix and the device was securely in place. More fluid was added to each balloon in turn, in 20 ml increments until each balloon contained 80 ml (maximum volume of fluid). Following this, clinical care was provided by clinical healthcare practitioners.

Women underwent monitoring of foetal condition and uterine activity by cardiotocography (CTG) according to the local protocol. CTG was discontinued once it was judged to be normal and the woman could go home. She was instructed to return to the hospital at an agreed time on the following morning, if the balloon catheter was spontaneously expelled or if she thought she was in labour, whichever was earlier. On the following morning/upon onset of labour, the device (Propess or CRB) was removed, and artificial rupture of membranes (ARM) attempted (unless spontaneous rupture had occurred already).

The intended recruitment target was randomisation of 120 women between the two sites over a 12-month period. Ability to recruit the intended sample size was considered the demonstration of feasibility.

### Sample size

The study was designed as a feasibility trial, and as suggested by NIHR guidelines [9], no formal sample size calculation was performed. It has been recommended that the total sample size for a feasibility trial can be between 70 (with allocation to treatments groups in a 1:1 ratio, i.e. 35 per group) if the outcome for the definitive RCT is normally distributed and a total of at least 120 subjects (60 per group) for binary outcomes [10]. It was important to allow for incomplete data and protocol violation. Therefore, it was thought more efficient to use the larger sample size to guard against the lack of precision by using inflated estimates. Therefore, the intended sample size would provide information on the primary outcome measures with sufficient accuracy to inform a power calculation for the definitive randomised controlled trial.

Women who declined to participate in the trial were invited to complete a short questionnaire exploring their main reasons for not participating. Verbal feedback was obtained for those who declined to complete the questionnaire. After they had given birth, the participants were asked to complete a questionnaire within 48 h. The questionnaire was modified from the one used in a previously published study [4]. This recorded women’s experience and acceptability of the two methods. All women who took part in the RCT were also invited to participate in a semi-structured interview at least 4 weeks after the birth. Partners were also invited with the women’s permission. The detailed methods and findings of the interviews as well as the post-natal questionnaire will be reported in a separate paper.

Participant demographics and clinical and patient-reported data were collected using an online database developed by KCTU. The clinical and patient-reported data included vital signs at trial entry, cervical Bishop score, birth details, maternal and foetal outcomes including adverse outcomes, use of pain relef measures at home and in the hospital, survey responses and decliner questionnaire responses.

The Adult Service Use Schedule (AD-SUS) was used to collect service use data [11]. It is a researcher-completed questionnaire, adapted for use in this study following a review of relevant and in collaboration with the clinical research team. The AD-SUS was completed using data from electroinc hospital records. Since this was a feasibility study, the usefulness of the instrument was judged by the ease of completion by the research staff and its ability to provide the data needed to generate costs for a full economic evaluation. The method for estimating the cost of the alternative interventions for this study required work in a feasibility stage because of the need to capture all aspects of induction. Service use data were reviewed in order to establish the most accurate approach to estimating the cost and alternative methods were compared. The options for sources of unit costs for the intervention and associated resources were also explored, making use of nationally available costs and optimising links with the service use data.

Research teams undertook standard assessments of safety, with reporting of adverse events and serious adverse events following usual governance procedures for a clinical trial of an investigational medicinal product overseen by the UK Medicines and Healthcare Regulatory Agency (MHRA).

### Statistical analysis

**Fig. 1:**
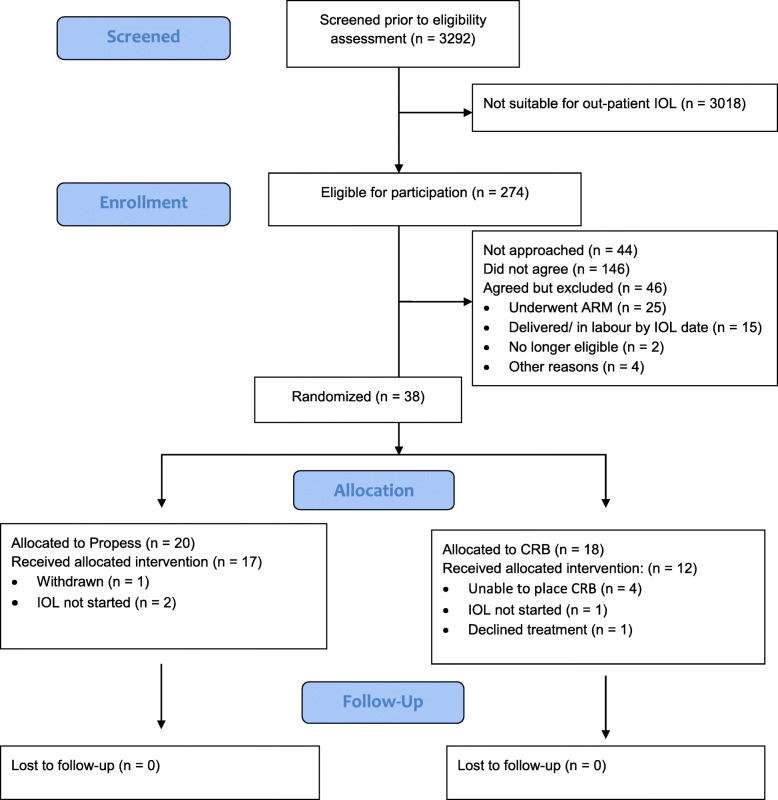
CONSORT diagram of the trial

The analysis and presentation of results follow the CONSORT guidelines [12] (Fig. 1). All analyses followed the intention-to-treat principle: all randomly allocated women (and infants) were analysed according to the group they were allocated to, irrespective of the intervention they received. Demographic and clinical data were presented as frequencies and percentages for categorical variables and mean and standard deviation for normally distributed continuous variables. Statistical hypothesis testing was not performed since this was a feasibility study.

### Role of funding source

The funder of the study had no role in study design, data collection, data analysis, data interpretation or writing of the report. The corresponding author had full access to all the data in the study and had final responsibility for the decision to submit for publication.

## Results

### Feasibility outcomes

Between October 16, 2017, and March 31, 2019 (18 months), 3293 women (2167 at St George’s and 1126 at Medway) underwent IOL. Out of these, 274 women (8.32%) were found to be eligible for inclusion according to local criteria for outpatient IOL. Of the 274 eligible women, 230 (83.9%) were approached for participation of whom 84 (36.5%) agreed (two/month/site). Of these, 46 women were excluded for reasons including ARM and delivery by IOL date. The remaining 38 women were randomised: Propess (*n* = 20), CRB (*n* = 18). Of the 146 women not agreeing to participate, 93 completed a questionnaire. Reasons for declining participation (please see Table 1) included declining IOL (*n* = 22), preference for inpatient IOL (*n* = 22) or for a specific method (Propess, *n* = 19). Of those randomised women, nine (24%) did not receive the intervention they were originally allocated (details in the Consort Flowchart). Feasibility outcomes according to the participating centre are shown in Table 2.

**Table 1:** Decliners’ information

	No. of women
Total women declining participation	117
Decliners’ data available	93
Verbal response	70
Decliners’ questionnaire	23
Main reason for declining	
Preferring inpatient IOL	22/93
Declining IOL by any method	22/93
Preferring Propess	19/93

**Table 2:** Feasibility outcomes by centre and for all participants

	SGH	Medway	All participants
Women delivering in the study period (*n*)	2167	1126	3292
Number of eligible participants (*n*, %)	168 (7.75%)	106 (9.41%)	274 (8.32%)
Number of participants approached (*n*, %)	156 (92.9%)	74 (69.8%)	230 (83.9%)
Participants randomised (*n*, %)	30 (19%)	8 (10%)	38 (16.5%)
Received allocated intervention (*n*, %)	23/30 (76.7%)	6/8 (75.0%)	29/38 (76.3%)
Did not receive allocated intervention (*n*, %)	7/30 (23.3%)	2/8 (25.0%)	8/38 (21.1%)
Withdrew from trial intervention (*n*)	0	1	1
Complete data available (*n*, %)	23 (76.6%)	5 (62.5%)	28 (73.7%)
Post-natal patient questionnaire completed (*n*, %)	23 (76.6%)	5 (62.5%)	28 (73.7%)
Declined post-natal patient questionnaire (*n*)	0	1	1
Agreed for post-natal interview (*n*)	14	7	21
Underwent post-natal interview (*n*)	14	7	21

*SGH* St. George’s University Hospital, London, *Medway* Medway University Hospital, Kent

### Clinical characteristics

Participant demographics at randomisation are shown in Table 3. The women had a mean height of 168.9 cm, mean weight 69.1 kg and mean BMI 24.2 kg/m^2^, whilst 25/38 (65.8%) were nulliparous. The majority of women 29/38 (76%) were of white European ethnicity. The mean age of women in the Propess group was 34.1 years, compared to 33.2 years in the CRB group.

**Table 3:** Demographics of randomised participants

Participant characteristics	Propess (***n*** = 20)	CRB (***n*** = 18)
Site		
SGH Medway	16 4	14 4
Mean age (SD) in years	34.1 (4.48)	33.2 (4.32)
Mean height (SD) in cm	166.62 (6.25)	171.59 (7.1)
Mean weight (SD) in kg	71.84 (11.9)	65.96 (11.0)
Mean BMI (SD) in kg/m^2^	25.72 (2.9)	22.37 (3.3)
Ethnicity		
White	15 (75%)	14 (78%)
Black	1 (5%)	2 (11%)
Asian	1 (5%)	0
Mixed	1 (5%)	2 (11%)
Other	1 (5%)	0
Not known	1 (5%)	0
Mean gestational week (SD) at delivery	41.71 (0.61)	41.63 (0.53)
Marital status		
Married	15 (75%)	7 (39%)
Cohabiting	4 (20%)	8 (44%)
Single	1 (5%)	0
Not recorded	0	3 (17%)
Employment		
Employed	16 (80%)	18 (100%)
Unemployed	2 (10%)	0
Not recorded	2 (10%)	0
Nulliparous	13 (65%)	12 (67%)
Parous	7 (35%)	6 (33%)

Data reported as mean (SD) or *n* (%)

Maternal and foetal parameters at baseline are shown in Table 4. Maternal vital signs were within the reference range at baseline, post-treatment and at follow-up. No uterine activity was detected at baseline in either of the two groups. The median Bishop score at study entry for both groups was unfavourable (Propess, 4; CRB, 3, Table 4). The clinical outcomes are shown in Table 5. The device (vaginal pessary or balloon catheter) was expelled in four (10.5%) of women. Seven of the 38 participants could not go home after intervention (Table 5). Epidural use for labour analgesia was reported by 20/38 (52.6%) of women. Nearly two thirds (61%) of the women in the cervical balloon group had a spontaneous vaginal delivery, compared to 45% in the dinoprostone group. Delivery was by caesarean section in 14 (36.8%) women. The rates of caesarean section were 33% (6/18) in the CRB group and 40% (8/20) the Propess group.

**Table 4:** Maternal and foetal clinical parameters at baseline

Parameter	Propess (***n*** = 20)	CRB (***n*** = 18)
Mean pulse (SD) in BPM	83.4 (10.35)	80.8 (9.63)
Mean systolic BP (SD) in mmHg	118.7 (9.71)	117.2 (12.40)
Mean diastolic BP (SD) in mmHg	76.9 (5.52)	77.3 (8.08)
Mean temperature (SD) in Celsius	36.7 (0.20)	36.6 (0.24)
Mean respiratory rate in breaths/min (SD)	16.4 (1.26)	16.1 (1.19)
Mean foetal heart rate (SD) in BPM	137.6 (12.18)	141.3 (12.83)
Median number (IQR) of contractions/10 min	0 (0 to 0)	0 (0 to 0)
Median Bishop score (IQR)	4 (3.0 to 4.0)	3.0 (2.8 to 4.3)

**Table 5:** Clinical and health economic outcomes

Participant outcome	Propess (***n*** = 20)	CRB (***n*** = 18)
Intervention expelled		
No	15 (75%)	14 (78%)
Yes	3 (15%)	1 (6%)
Missing	2 (10%)	3 (16%)
Return for admission		
Agreed time next morning	5 (25%)	4 (22%)
Labour	10 (50%)	5 (28%)
Participant never went home following intervention	3 (15%)	4 (22%)
Missing	2 (10%)	5 (28%)
Epidural use		
No	8 (40%)	9 (50%)
Yes	12 (60%)	8 (44%)
Missing	0	1 (6%)
Live birth	20 (100%)	18 (100%)
Mode of birth		
SVD	9 (45%)	11 (61%)
Caesarean section in labour	6 (30%)	2 (11%)
No labour caesarean section	2 (10%)	4 (22%)
Instrumental delivery	3 (15%)	1 (6%)
Median estimated blood loss in ml (IQR)	320.0 (200.0 to 675.0)	600.0 (225.0 to 1145.0)
Mean birthweight in gm (SD)	3688.7 (310.00)	3684.2 (293.33)
Boy	14 (70%)	6 (33%)
Girl	6 (30%)	11 (61%)
Not recorded	0	1 (6%)
5 min Apgar score median (IQR)	10.0 (10.0 to 10.0)	10.0 (10.0 to 10.0)
Head circumference in cm (mean, SD)	35.2 (1.54)	35.1 (1.36)
Where did the baby go?		
To mother	20 (100%)	16 (89%)
NICU admission	0	1 (6%)
Missing	0	1 (6%)
**Any adverse event experienced**		
Mother alone	8 (40%)	6 (33%)
Baby alone	1 (5%)	3 (17%)
Both mother and baby	4 (20%)	2 (11%)
None	7 (35%)	7 (39%)
**Severe adverse event**		
None	18 (90%)	17 (94%)
Mother alone	1† (10%)	1 (6%)
Baby alone	0	0
Both mother and baby	0	0
**Maternal death**	0	0
**Foetal/neonatal death**	0	0
**Health economic outcomes**	**Propess (*n*** **= 19)**	**CRB (*n*** **= 17)**
Cost of induction and readmission prior to delivery, mean (SD)	135.47 (206.56)	127.29 (198.33)
Cost of delivery, mean (SD)	3254.16 (965.10)	2753.53 (712.31)
Total cost, mean (SD)	3389.63 (1023.94)	2880.82 (717.04)

†One mother experienced two severe adverse events (haemorrhage and infection)

Median gestational age at delivery was 41^+6^ weeks. Mean birthweights were similar between groups (Propess, 3688.7 gm; CRB, 3684.2 gm). All babies except one (37, 97.4%) remained with the mother after delivery. No maternal or foetal deaths were recorded in this small sample.

The health economic outcomes are as follows: the AD-SUS service use questionnaire was easy to complete from patient records and the completeness of the data was excellent; 95% of participants had full service use data available at follow-up. Close monitoring over the data collection period ensured that we are confident that all relevant resources were included in the analysis. Full data at follow-up were available for 36 of the 38 randomised women. The costs of induction and readmission prior to delivery were estimated to be similar between the two randomised groups. Total costs were £2880.82 in the CRB group and £3389.63 in the Propess group (please see Table 5). This difference was accounted for by differences in mode of birth and it cannot be assumed from this small sample that a difference in mode of birth would be found with bigger numbers.

### Women’s willingness to be randomised

Data were collected on reasons for declining participation. Overall, 93 women supplied decliners’ data with 23 women completing a decliner’s questionnaire and 70 women providing verbal responses. The most common reasons for declining to take part were as follows: preferring to have inpatient IOL (*n* = 22; 24%), declining IOL by any method before 42 weeks (*n* = 22; 24%) and preferring to have PGE_2_ pessary (*n* = 19; 20%).

## Discussion

The number of women eligible for outpatient induction was much lower than anticipated. Reasons for under-recruitment included understaffing and not having the midwife(s) available to screen women every day and women’s preference to a method, in particular prostaglandin pessary. Suitability of outpatient induction is dependent on the local criteria. Units with restrictive criteria will have a limited number of women deemed suitable for outpatient induction of labour. A widening of inclusion criteria may increase numbers of potential participants. The study is not feasible using existing criteria and further modifications to the eligibility criteria for outpatient IOL would be needed to make an RCT feasible. At Medway Hospital, consenting women underwent an artificial rupture of membranes (ARM) if feasible rather than entry into the trial due to a policy change. This led to a reduction in the pool of possible participants limiting recruitment. Therefore, further recruitment at Medway Hospital was stopped. Choice of another suitable unit where consenting women were allowed to participate in the study could have helped with recruitment.

Approximately a third of all eligible women in this study (84/230, 36.5%) were prepared to participate in a trial where the method of induction of labour in the outpatient setting (dinoprostone or CRB) is chosen at random. Participant numbers were limited further by clinical factors, particularly women going into labour spontaneously before the CR method was inserted, and a smaller number of women withdrawing after recruitment. A previous study by Henry et al. [4] exploring outpatient Foley catheter versus inpatient prostaglandin E2 gel for induction of labour reported that out of the 262 women found eligible for inclusion, 101 (38.5%) agreed and were randomised. This rate is very similar to that observed in this study, although the Henry study took place in Australia.

Outpatient induction of labour is not common in the UK. In a survey of outpatient IOL [13], a postal questionnaire was sent to 210 NHS consultant-led obstetric units within the UK, of which 78% responded. Only 17.6% of units reported that they currently or soon will provide outpatient IOL. The rate of use may have increased since this survey but routine data are not available. Outpatient IOL may benefit the working of midwives as well. A survey exploring the impact of outpatient IOL on midwives’ work found that their job satisfaction either increased or was unchanged in an overwhelming majority (93%) of respondents [14].

More than one half of those women approached in this study declined to participate. The most common reasons for declining to take part were preferring to have inpatient IOL, declining IOL by any method before 42 weeks, plus preferring to have PGE_2_ pessary. Since a trial of this nature cannot be blinded, there is also a possibility that agreement to continue in a trial may be skewed by women’s prior attitudes. This is reflected in the reasons given for declining, and CRB was not standard practice in these services; preference for this method could equally have been a motivator for participation. Mechanical methods have been reported as being safer than prosaglandins for labour induction [15]. However, that study was published relatively recently and the findings not widely disseminated. Improved information provision may remove this obstacle and provide eligible women with a wider choice.

A majority of women who were randomised received the intended intervention and were able to go home. Delivery was by caesarean section in 14 (36.8%) women. All babies were born with good condition and only one baby did not stay with the mother after delivery. Severe complications were reported only in a small minority (Table 5). One woman from the CRB group experienced severe post-partum haemorrhage (2.1 L) and one woman from the Propess group underwent a category 1 caesarean section for antepartum haemorrhage.

Not all women who desired to go home could go home. Twenty-nine percent of the participating women could remain home overnight in this study. Wilkinson et al. [7] reported that less than half the women participating in an RCT comparing inpatient versus outpatient cervical ripening could remain at home overnight. However, all women had received vaginal prostaglandin E_2_ gel [7].

## Conclusions

The study is not feasible using existing eligibility criteria for outpatient induction of labour (IOL). Further modifications to the eligibility criteria for outpatient IOL improved provision of information on safety and better availability of research staff may be helpful to make such a trial feasible. The trial procedures were acceptable to women who participated. Although the sample size of this feasibility trial was limited, no major adverse effects attributible either to the setting or the method for induction of labour were recorded. Service use data can be collected in a vast majority of participants.

## Data Availability

The trial essential documents along with the trial database will be archived in accordance with the Sponsor (Joint Research and Enterprise Office, St. George’s, University of London) SOP JREOSOP0016. The agreed archiving period for this trial will be 15 years. The data will be available for sharing by contacting the chief investigator. Publication policy has been documented in the project protocol.
